# 24402 Grounded Theory Model for Adherence to Home Exercise among People with a Mobility Disability

**DOI:** 10.1017/cts.2021.573

**Published:** 2021-03-30

**Authors:** Jereme Wilroy, Ivan Herbey, Nataliya Ivankova, James Rimmer

**Affiliations:** University of Alabama at Birmingham

## Abstract

ABSTRACT IMPACT: Future exercise interventions for people with mobility disability should be tailored to the level of the individual and responsive to intervention adherence. A promising area of research is the use of adaptive interventions as a mechanism for tailoring and 'titrating’ the intervention based on data obtained during early intervention stages. OBJECTIVES/GOALS: The purpose of the study was to understand adherence to a home-based exercise trial delivered to people with a mobility disability. The objective was to develop a multi-dimensional model for engaging and retaining participants not adhering to an exercise trial by using a grounded theory approach involving both qualitative and quantitative data. METHODS/STUDY POPULATION: An exercise trial utilized telehealth technology to deliver a home-based exercise intervention for adults with a mobility disability. In order to understand factors of adherence to the exercise trial, a mixed methods study design was used involving baseline data and semi-structured interviews. Maximum variation sampling was used to select participants based on level of adherence, gender, race, and functional level. Categorization based on adherence to exercise videos during the first 12 weeks of the intervention included 3 groups: 1) high adherence (≥80% weekly median exercise video minutes viewed), 2) sub-optimal adherence (< 80% but ≥20%), and 3) low adherence (< 20%). Interviews were conducted with 10 participants in each group (n = 30) and data were analyzed using a grounded theory approach. RESULTS/ANTICIPATED RESULTS: A sample of 30 participants from a large pragmatic, home-based exercise trail have completed interviews. All interviews were transcribed and uploaded to NVivo software for coding. Emerging codes include lack of time to exercise, inappropriate exercise intensity, and lack of support for exercise. Using grounded theory approach, results include: 1) identifying risk factors for low adherence to a home-based exercise program delivered to people with mobility disability, 2) discovering themes for not responding to program activities in a home-based exercise program for adults with physical disabilities, and 3) determining the relationships between variables that emerge from thematic and statistical analyses. A model for adherence to home exercises among people with mobility disability will be presented. DISCUSSION/SIGNIFICANCE OF FINDINGS: People with a mobility disability are more susceptible to adopting sedentary lifestyles, which result in poor psychosocial and physical health outcomes. There is a clear and pressing need for designing future home-based exercise interventions with a greater level of customization for participants who have low to non-adherence.

